# Early labor market experience and preferences for social spending: the impact of occupational exposure to foreign demand

**DOI:** 10.3389/fsoc.2025.1577352

**Published:** 2025-06-18

**Authors:** Jérémie Poltier

**Affiliations:** Department of Political Science and International Relations, University of Geneva, Geneva, Switzerland

**Keywords:** political economy, redistributive preferences, exposure to foreign demand, competitiveness concerns, early labor market experience, workplace socialization

## Abstract

An individual's early labor market experience and the associated workplace socialization constitute an important time in a person's life. During those years, the labor market entrant interacts with her superiors, her senior colleagues and with her firm's broader ecosystem. During this process, she is exposed to discourses that emphasize the firm's interests, as well as a narrative that tends to conflate the firm's interests and the interests of its employees, which likely contributes to shape her economic policy preferences. This paper asks the following questions: how does early socialization in the workplace contribute to shape economic attitudes, and does it have a lasting impact on these attitudes? Using data from the Swiss Household Panel, I investigate the trajectory of young individuals during their early years on the labor market to gain insight about the interplay between occupational characteristics and economic attitudes. In line with recent findings in the trade exposure literature that emphasize the association between exposure to foreign demand and concerns over international competitiveness, I find that labor market entrants in occupations associated with export-dependent sectors develop hostile preferences vis-à-vis social spending. Taking advantage of the panel structure of the data, I show that while individuals do self-select into occupations based on pre-existing preferences—confirming that the self-selection mechanism is relevant to the occupation-preferences link—, they also adjust these preferences as a result of their early labor market experience—confirming the importance of the socialization mechanism.

## 1 Introduction

Several strands of literature highlight the relationship between various dimensions of an individual's position in the labor market and her economic policy preferences. These dimensions include occupational class (Oesch, 2006; Kitschelt and Rehm, 2014), income and exposure to different forms of risk (Iversen and Soskice, 2001; Moene and Wallerstein, 2001; Rehm, 2009). Within the latter category, one strand of literature focuses on the link between an individual's exposure to international competition and her preferences for insurance and compensatory social policies.

The received wisdom of the “compensation hypothesis” is as follows. Increased exposure to international competition entails a greater degree of exposure to risk due to the volatility of international markets, and exposure to risk makes individuals more likely to support compensatory and insurance policies, as they realize that they may need to rely on such policies (Balcells Ventura, 2006; Walter, 2010). However, recent research has found that this is often not the case, with increased exposure to international competition often associated with lower demands for compensation and anti-redistributive preferences (Colantone and Stanig, 2018a,b; Wren and Rehm, 2014; Poltier, 2025). The dominant explanation for this phenomenon is that the acceleration of globalization has led to an increase in the economic costs of compensation and that citizens have become increasingly cognizant of this reality (Steinmo, 1994; Rodrik, 1997). Colantone and Stanig (2018a,b) contend that electorates have largely internalized the constraints weighing down on compensatory policies, fostering a rise in *economic nationalism*—a mix of domestic free-market policies and protectionism—, particularly in regions adversely affected by Chinese import penetration. A neighboring perspective highlights instead that individuals associated with sectors that are heavily reliant on their ability to export adopt anti-redistributive preferences due to concerns that redistributive policies might negatively impact the country's international competitiveness. Wren and Rehm (2014) show for example that skilled individuals working in export-oriented sectors are especially hostile to compensatory social policies, despite being fairly exposed to risk themselves. Finally, Poltier (2025) analyzes the impact of regional export-dependence on the outcome of business-related popular votes across Swiss employment zones and finds that rising export-dependence is associated with electorates becoming more aligned with the (anti-redistributive) vote recommendations of the country's peak business interest association.

A key weakness of the growing literature pertaining to the individual-level link between exposure to international competition and economic preferences is that it has so far relied mostly on cross-sectional studies. While these are important in their own right, they fail to account for the possibility that individuals may self-select into occupations with certain characteristics based on their prior preferences, by opposition to being socialized into these preferences through their occupation. Kitschelt and Rehm (2014) discuss the self-selection vs. socialization question with respect to the broader link between occupation and preferences and argue that the two mechanisms need not be exclusive to one another, adding that both mechanisms likely contribute to the observed link. Somewhat related to the self-selection vs. socialization question, early occupational choices as well as early labor market experience typically occur during what has been coined by some as the impressionable years, a period during which susceptibility to changes in attitudes is much higher than later in life (Krosnick and Alwin, 1989). Moreover, research has shown that one's economic experience during early adulthood can have a long-lasting impact on political attitudes (Krishnarajan et al., 2023), as well as political interest and participation (Emmenegger et al., 2017).

I thus use an original empirical strategy relying on the Swiss Household Panel (SHP Group, 2024), focusing primarily on new labor market entrants, that is to say young people that proceed to start their first job following their education. The strength of this approach is that it enables me to observe an individual's preferences for federal social spending—my main dependent variable of interest—prior to them making their first entry into the labor market, and compare these to their preferences after several years spent working. Most importantly, my design makes it possible to assess the effect of occupational exposure to foreign demand on preferences for social spending, controlling for pre-entry preferences. I can thus investigate the importance of early workplace socialization in shaping economic policy preferences, while also testing whether individuals self-select into occupations with certain characteristics based on their pre-entry preferences. Occupational exposure to foreign demand constitutes my main independent variable of interest. Drawing on the methodology of Baccaro and Neimanns (2022), it is an occupation-level measure that aims to integrate information about the sectors in which an individual with a given occupation is likely to be active, as well as how exposed these sectors are to foreign demand.

To briefly anticipate the main findings of the article, I find that an individual's preferences prior to entering the labor market are indeed associated with the characteristics of their occupation after a few years on the labor market, which suggests that self-selection does occur. Most importantly, however, I show that an individual's occupational exposure to foreign demand after a few years on the labor market has a significant negative impact on her support for social spending, even when controlling for the preferences she held prior to entering the labor market. By contrast, changes in occupational characteristics later in life are shown to have no impact on redistributive preferences. Overall, my findings suggest that while young individuals do self-select into occupations based on their redistributive preferences, occupational characteristics impact redistributive preferences beyond what can be explained through self-selection.

This article thus provides evidence in favor of both the self-selection and the socialization mechanisms with respect to the link between occupation and preferences, highlighting in particular the importance of socialization during the years following labor market entry. In itself, this constitutes an important contribution to several literatures across both sociology and political science, which concern themselves with this link and sometimes struggle with problems of endogeneity. By assessing this two-way causal link between occupational exposure to foreign demand and social spending preferences, the article provides additional evidence that the posited mechanism hinging on concerns over competitiveness is indeed a relevant one. This constitutes a meaningful contribution to the trade exposure literature.

Section 2 presents the article's theoretical framework, reviews the literature on trade exposure, and discusses the rationale for studying new labor market entrants to address my questions of interest. It then formulates testable hypotheses about the main association of interest as well as potential conditioning factors. Section 3 briefly introduces the article's research design and data sources, before going into more detail about the article's empirical strategy. Section 4 presents three sets results: one pertaining to the general working population, one to the problem of self-selection and one to the impact of early workplace socialization. Section 5 offers a conclusion.

## 2 Theoretical framework

### 2.1 Exposure to foreign demand and economic policy preferences

As alluded to in the introduction, recent findings in the trade exposure literature have called into question the received wisdom of the “compensation hypothesis,” with several scholars arguing that the scope conditions for its validity no longer exist in developed economies. The intuition behind the “compensation hypothesis” proceeds in two steps, a first one that identifies the losers or potential losers of increased trade openness, and a second one that makes an argument about the social policy preferences of individuals identified as such. Increased trade openness, though generally thought to deliver increased standards of living in the aggregate, tends to be accompanied by negative aspects. It generates increased economic volatility and thus uncertainty, and also creates losers, namely people who are worse off as a consequence of an increase to trade openness. Different families of economic models of trade predict which specific group will lose out from freer trade, with either education/skill or industry presented as the main factor (see Scheve and Slaughter, 2001; Hays et al., 2005; Walter, 2010 for an overview of these models). In the second step, the proponents of the “compensation hypothesis” argue that those at risk of becoming losers of globalization desire the adoption (or reinforcement) of policies that would compensate them in the event of job (and thus income) loss, such as unemployment insurance or other redistributive and compensatory policies.

Among the two steps described above, the one that has been the target of criticism is primarily the second one. Indeed, scholars have argued that in a globalized economy, generous compensatory policies are likely to be associated with decreased international competitiveness (Steinmo, 1994; Alesina and Perotti, 1997; Rodrik, 1997). Lower competitiveness, in turn, is especially detrimental to sectors that compete with foreign firms, be it as exporters competing for global market share or as domestically-oriented producers competing against imported goods and services. For workers affiliated with these sectors, lower demand means at best fewer advancement opportunities and poorer overall working conditions, at worst job loss or pay cut. In that view, when the firm loses out, so too does the worker, and a worker may be convinced that fostering conditions that favor her firm's ability to compete internationally is more important than hedging her bets against the risk of job loss.

Policy discourses on the importance of competitiveness have flourished since the 1990s, including the idea that nation states should use all levers at their disposal to foster a more competitive economy. This carries implications for a whole series of policy domains related to the workforce, the labor market, infrastructure and of course welfare generosity (Cerny, 1997; Fougner, 2006; Linsi, 2020). The success of these discourses in policymaking circles leads to their normalization over time, and to the ideas they carry being at least in part internalized by the broader public, affected workers and citizens alike. The mechanism proposed in this article to explain a negative association between exposure to foreign demand and preferences for social spending are as follows. While I argue that the general idea that welfare generosity is harmful to international competitiveness is likely to be widespread among the population, I expect individuals with a higher occupational exposure to foreign demand to be more *concerned* about competitiveness than their less exposed counterparts. As a result, a new entrant in a highly exposed occupation is not only more likely to hear discourses about the association between welfare generosity and competitiveness, these discourses are also more likely to be framed as being about *her* and *her* interests, and I expect her to adjust her preferences for social spending as a result of this process.

### 2.2 Why study new labor market entrants?

One important reason to focus exclusively on new market entrants has to do with the problem of self-selection. If individuals self-select into occupations with specific characteristics based on prior preferences, then the validity of any inferences about the impact of occupational characteristics on preferences is threatened. In the case at hand, it is indeed plausible that prior economic preferences inherited through her early socialization may affect an individual's early choices in the labor market. If individuals who inherited more hostile preferences toward social spending and redistribution through their early socialization tend to opt for occupations associated with export-dependent sectors, then at least a portion of the association measured in cross-sectional studies stems from a causal relationship that is the reverse of the relationship posited by the theoretical framework highlighted thus far. Moreover, this is true even if the locus of occupational choice does not directly factor in export-dependence as such, but instead pertains to the “work logic” associated with a given occupation (Oesch, 2006; Kitschelt and Rehm, 2014). To give an example of how self-selection may apply to the issue at hand using two occupations that are polar opposites on the exposure to foreign demand dimension, it seems highly plausible that, even before coming into contact with the labor market, individuals who later become health care assistants view social spending more favorably, on average, than those who later become mechanical engineering technicians.

While addressing the question of self-selection constitutes an important reason to focus on the years surrounding an individual's entry into the labor market, it is not the only one. Work occupies a hugely important place in a person's life, be it in terms of the sheer amount of time that an average individual spends at work over her lifetime, or by virtue of it being a highly significant vector of socialization. As such, the years during which an individual transitions into the world of work constitute a highly consequential moment, one that is likely to have a lasting impact on her. Research has also shown that firms tend to provide more structure for the onboarding of new workers transitioning from school (Bauer et al., 2007; Ashforth et al., 2007; Saks and Gruman, 2012). During those years, the labor market entrant interacts with her superiors as well as senior colleagues, and more generally with her firm's ecosystem. During this process, she learns more about her particular occupation and how it relates to her firm and economic sector. She is also exposed to discourses pertaining to how her firm or other firms in related sectors interact with their broader environment, both domestic and international. This entails the firm's relationship with its clients, its suppliers, as well as the broader economic and regulatory environment. In other words, through this process of workplace socialization, the new labor market entrant becomes exposed to a point of view that emphasizes the firm's interests, as well as a narrative that tends to conflate the firm's interests and the interests of its employees. My core hypothesis is that coming into contact with this point of view and discourses has a significant impact on the economic policy preferences of new labor market entrants, leading them to adopt preferences that are aligned with the perceived interests of their firm or sector.

While the “new job experience” of older workers may in certain ways be similar to the process described above, the fact that they have had previous labor market (and life) experience does constitute a meaningful difference. The years that cover the period of early adulthood are sometimes known as the *impressionable years*, that is to say a period during which individuals are highly susceptible to attitudinal changes, which become significantly less likely later in life (Krosnick and Alwin, 1989). Research has also shown that economic experiences during one's youth can have a lasting impact on attitudes. Krishnarajan et al. (2023) show that people who had positive (negative) economic experiences during their early adulthood are significantly more (less) supportive of the type of regime in which they live, be it an authoritarian regime or a democratic one. In an analogous vein, Emmenegger et al. (2017) show that for young workers, the impact of unemployment spells on political interest and participation is strong, with scarring effects still present more than a decade after the experience of unemployment. By contrast, the impact of unemployment spells on these dimensions is much more limited for older workers, as their political habits are already well-formed. Wehl (2023) finds similar results with respect to unemployment and redistributive attitudes. This is not to say that older workers never adjust their preferences to the changes they experience in their working lives. Rather, the idea is that their preferences become more rigid and harder to move. They are unlikely to respond to incremental changes and instead require a stronger stimulus or shock to be moved.

### 2.3 Formulating hypotheses

Applying these insights to the question of interest, my assumption is that one's early experience in the labor market tends to “leave its mark” and have a lasting influence. Of course, first time labor market entrants arrive on the job with a set of pre-existing dispositions, but for them, the world of work constitutes a new dimension of socialization. Insofar as economic policy matters are relevant to how they interact with their work, their economic policy preferences ought to be relatively malleable with respect to this new dimension of socialization. A likely outcome is thus for them to adjust their preferences in the direction of the dominant view held within their new professional environment. By contrast, an older worker who switches from one occupation to an entirely different one is less likely to adjust her preferences as a result, as these have become more firmly established throughout her life (and work) trajectory. The above constitutes the foundation for my baseline expectation that *new labor market entrants in occupations that are strongly associated with export-dependent sectors develop hostile preferences vis-à-vis social spending as a result of workplace socialization* (H1). The baseline self-selection hypothesis, conversely, would be that *individuals with hostile prior preferences vis-à-vis social spending are more likely to choose occupations with a strong exposure to foreign demand* (H2). On the other hand, I expect that *among older workers, changes in occupational characteristics are unlikely to trigger changes in redistributive preferences* (H3).

When it comes to factors that could explain variation with respect to H1, the literature on organizational socialization in the field of management studies can be of inspiration (Wanberg, 2012). A key focus of this literature is to explain variation in “organizational commitment” across different kinds of workers within different kinds of organizations, and subsequently derive best practices to foster such commitment among workers. With respect to my research question, I argue that workers more committed to their organization are most likely to adjust their preferences according to their firm or sector's interest, so that factors found to foster organizational commitment are likely to reinforce my relationship of interest. Job satisfaction is one of the most important drivers of organizational commitment highlighted in this literature (Gruman et al., 2006), so that *I expect job satisfaction to reinforce the negative association between occupational exposure to foreign demand and preferences for social spending* (H1a). Education is another potential moderator, although there are grounds to make competing predictions. On the one hand, attending university represents a transformative experience for many individuals, one that takes place during the *impressionable years* and where individuals develop their identity and preferences, including political and economic preferences. University-educated individuals also tend to have more general skills, which could lead them to be less attached to a specific job or occupation and thus less worried about the prospect of job loss. As a result of the above, one might therefore *expect the adjustment of preferences to be stronger among non-university-educated new labor market entrants* (H1b). On the other hand, *there are reasons to expect that university-educated entrants tend to be more personally invested in their job, due to their job being more interesting or fulfilling, which would lead one to expect a stronger process of preferences adjustment* (H1c). With respect to self-selection, there are good reasons to expect that, owing to more firmly established preferences, *the importance of self-selection based on prior preferences is stronger for university-educated new entrants* (H2a). Furthermore, this is likely to be amplified by university-educated new entrants having more job opportunities and thus more agency to actually self-select into desired occupations and sectors. Union membership is another relevant dimension, as unions can be an important alternative vector of work-related socialization. Union members are bound to be heavily exposed to the discourses of union representatives, so the tonality of this discourse is key to formulating expectations. Highly specialized unions associated with a particular economic sector may promote a discourse that emphasizes the importance of competitiveness, in which case *the relationship between occupational exposure and anti-redistributive preferences would be reinforced by union membership* (H1d). Alternatively, encompassing unions that organize a broad range of workers are more likely to have a discourse that focuses on solidarity and, more relevant to union membership as a moderating variable, one that prioritizes the logic of the compensation hypothesis—exposure is associated with heightened risk for the worker and thus heightened demand for compensatory measures—over that of international competitiveness. This would lead one to expect *occupational exposure to foreign demand to have less of a negative impact on redistributive preferences among new labor market entrants who are union members* (H1e).

## 3 Materials and methods

### 3.1 Research design

**Figure 1:**
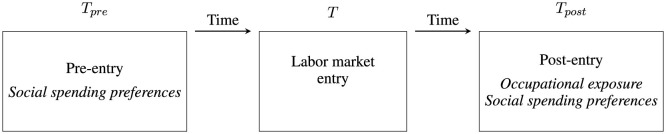
Schematic representation of research design.

To test the hypotheses outlined above, I need to observe new entrants to the labor market at three different points in time, which are summarized in Figure 1. First, I need to observe the individual's entry into the labor market, namely the point in time that serves to anchor the other two moments of interest. I call this *T*. Then, I need to observe the individual's preferences for social spending prior to her entry into the labor market, that is, at a time when she has not yet entered into an occupation or experienced any kind of workplace socialization (*T*_*pre*_). Finally, I need to observe the same preferences again after the individual has undergone several years of workplace socialization, at which point I also need to observe the characteristics of her occupation in terms of exposure to foreign demand (*T*_*post*_). Figure 1 is a schematic and abstract representation of my research design. As will become clear in the following subsections, its application to real data requires some flexibility.

### 3.2 Data sources

The primary data source for this paper is the Swiss Household Panel (SHP Group, 2024), a longitudinal social science survey that has interviewed members of private households in Switzerland since 1999^1^. As I discuss below, I rely on this data source to identify young entrants to the Swiss labor market and to follow their trajectories from the years before their entry to several years after their entry, when they have accumulated meaningful work experience. In order to compute my independent variable of interest, occupational exposure to foreign demand, I further rely on waves 1 through 10 of the European Social Survey (European Social Survey, 2020) and the Trade in Value Added database from the Organization for Economic Cooperation and Development (OECD, 2023). I use the former in order to obtain data on how occupations are distributed across economic sectors and the latter for my sectoral quantity of interest, Domestic Value Added embodied in Foreign Final Demand (FFDDVA) (for details, see Section 3.4).

### 3.3 Identifying new labor market entrants

There are no perfect ways to identify new labor market entrants throughout the SHP. One of the core ideas is to identify when respondents are observed as “actively occupied” for the first time, after having been observed as outside of the labor force at least once prior. This process is arguably sufficient to capture the first-time labor market entry of young individuals who have long (passively) been a part of the SHP through their household's participation. For those who satisfy this condition as newer participants to the panel, it is possible to exclude those who declare having previously held a job. There is, however, an additional choice to make, since a significant proportion of young respondents declare to be “actively occupied” at the same time as they declare pursuing their initial training, be it dual-track vocational training, vocational high school or university, among other options. This poses the underlying question of what constitutes labor market entry properly speaking. When a student holds a part-time job, that job is unlikely to be representative of her future labor market position, be it in terms of the tasks or the type of workplace socialization associated with the job. It is therefore generally safe to assume that there is something qualitatively different about her post-study labor market entry. On the other hand, for those pursuing a dual vocational training track, the in-firm part of their training tends to be fairly representative of their future labor market position, in terms of both tasks and workplace socialization.

Based on the above, the main results presented below consider the *post-studies* labor market entry of those respondents who follow strictly school-based training, including those who follow a full-time vocational school training. For respondents whose training includes in-firm training, I allow labor-market entry to be defined by the “actively occupied” status. Finally, although the aforementioned criteria already do much to exclude older respondents from the sample, it is important to set a maximum limit for the age of the respondent in *T*, the year of their labor-market entry. For the main results, I exclude respondents who are older than 30 years old in *T*. In addition, I exclude all respondents who are younger than 18 years old in *T*_*post*_, as I am primarily interested in the economic policy preferences of individuals who have reached the age of majority. Section 4.4 explores the impact of altering the labor-market entry criteria defined above, including disregarding the in-training restrictions, setting different age thresholds and setting a minimum earnings threshold.

### 3.4 Main IV of interest: occupational exposure to foreign demand

I measure occupational exposure to foreign demand using a measure inspired by Baccaro and Neimanns (2022). As stated in the introduction, the general principle of this method is to measure a sector-level quantity of interest at the occupation-level. This quantity of interest is Domestic Value Added embodied in Foreign Final Demand (FFDDVA), as a share of total value added, which Poltier (2025) uses to construct his measure of export-dependence. The idea is to create a more fine-grained measure of FFDDVA, which reflects differences across sectors in terms of that variable, as well as differences across occupations in terms of their pattern of association with specific sectors. An important rationale for the development of the method lies in the fact that precise measures of occupation (for instance ISCO at the 4-digits level) are highly common in survey data, whereas data on economic sector or activity tend to be quite rough, often corresponding to the first level of NACE^2^. Most importantly, in the first level of NACE, manufacturing is treated as a single bloc, and the diversity of the manufacturing sector is only visible with the second level of NACE. Fortunately, the European Social Survey (European Social Survey, 2020) contains information on both the respondent's occupation (ISCO-4-digits^3^) and economic sector (NACE-2-digits), which allows me to map how occupations relate to sectors and thus estimate the probability that an individual with occupation *i* will be active in sector *j*. These probabilities can then be used as weights to distribute the measure of exposure to foreign demand from the country-sector-year level down to the level of occupations. The specifics of the procedure are described in the Supplementary material. This method leverages variation at both the sector (in terms of FFDDVA) and occupation-level (in terms of probability to be employed in sector *j*) in order to create a fine-grained measure of occupational exposure to foreign demand. The measure generated by this method is substantively meaningful in that it faithfully reflects an individual's opportunity structure across sectors, given her occupation. To give an example, this accounts for the fact that the opportunity structure—and thus the perceived interest—of Accounting and Bookkeeping Clerks across economic sectors ought to be quite distinct from that of Rubber Products Machine Operators, even if they find themselves working in the same sector or even the same firm at a given point in time.

### 3.5 Dealing with uneven availability in the DV

My key dependent variable of interest is based on an item that asks respondents whether they want to see federal social expenses decrease, stay the same, or increase. This is meant to capture support for redistributive social policies. My preferred approach is to use a dichotomized version of the variable identifying respondents who specifically support an increase in spending, but I also report results for a continuous coding of the variable. Importantly, the item is available for years 1999 through 2009, and subsequently in 2011, 2014, 2017, and 2020, as part of the “political behavior and values” module of the SHP. Coupled with missingness due to occasional non-response, this means that in the second half of the time period covered, data on preferences become relatively sparse.

In order to address the issue, my strategy is as follows. As mentioned above, I define three time points for each respondents: a pre-entry time point (*T*_*pre*_), the time point of labor-market entry (*T*), and finally the post-entry time point (*T*_*post*_). *T* simply refers to the time point defined in Section 3.3 above, or variations thereof in the case of robustness tests. *T*_*pre*_ and *T*_*post*_ are more constrained, in that preferences must be observed at both of these time points. For both *T*_*pre*_ and *T*_*post*_, I rank the different possibilities in terms of distance to *T*, going from what I consider to be the ideal distance to less than ideal but still acceptable distances. For *T*_*pre*_, I consider the ideal distance to be *T*−1, then *T*−2, and so on up to *T*−4. For *T*_*post*_, I consider the ideal distance to be in *T*+3, followed by *T*+2, *T*+4 and finally *T*+1. Adopting such a flexible approach is a necessity considering the above mentioned gaps in the data, which compound with expected missingness patterns. Note that the respondent must be active in the labor market in *T*_*post*_, since exposure must be observed at the same time as preferences, and integration into the labor market is a necessary condition for the hypothesized mechanism to operate. Section 4.4 returns to this requirement and its impact on the results, while I examine the issue of dropouts from the main sample in more details in the Supplementary material.

Following the rules above, I obtain a main sample of 921 unique individuals, each observed at three different points in time, which form the baseline sample. Supplementary Figure S1 presents histograms of the distribution of *T*_*pre*_ and *T*_*post*_ in terms of absolute distance to *T*, and Supplementary Table S3 gives information about the distribution of *T* and *T*_*post*_ years in the final sample. As could be expected, there are fewer suitable cases after 2009, and these can also be expected to be different in terms of their effective distance to *T*. Section 4.4 discusses whether this affects the results.

### 3.6 Control variables

The results section below includes different analyses with different requirement in terms of control variables. For example, in the case of the analyses aiming to test the socialization hypotheses (H1 and H1a through H1e), there is a question as to whether some of the controls that one may be tempted to add would be so-called “bad controls.” For instance, considering the fact that I will be including pre-entry preferences as a control in most of my models, the inclusion of controls that are inherently stable (or merely tend to be stable between the pre- and post- time points) are not warranted. For example, female respondents tend to be more supportive of redistributive policies, but since pre-entry preferences already factor in the gender of the respondent, this particular analysis of new labor market entrants should not include such a control. A similar reasoning goes for Swiss citizenship. This does not pertain to the other analyses, which include gender as a control variable.

In the main analysis, other control variables are measured at the same time point as the DV, in *T*_*post*_. Among these, I include a measure of logged personal work income, which is probably the most relevant measure of income in this context. Using household income instead would lead to some difficulties. For one thing, some of these first-time labor entrants may still be living with their parents, while others may not. In addition, the effect of (primarily parental) household income on their social policy preferences is most likely already absorbed by their preferences in *T*_*pre*_. I therefore argue that any differences in preferences between *T*_*pre*_ and *T*_*post*_ associated with income matters are likely to be about personal work income. In addition to the previously mentioned moderator variables—job satisfaction, university education, and union membership—I also control for age in *T*_*post*_. Some controls are specific to the self-selection analysis, such as parental social class and a dummy indicating whether the respondent lived in a German-speaking household before entering the labor market. As for Swiss citizenship and public sector employment, I only include them in my large-N analysis of older workers. As 96% of respondents in my smaller sample are Swiss citizens, there are simply too few non-Swiss citizens to include this variable in the self-selection analysis. Regarding public sector employment, the variable does have a relatively high number of missing cases, so rather than including it as part of my main analyses, I evaluate its impact as a robustness test.

Table 1 presents a few descriptives pertaining to the various variables mentioned above. It shows that support for increased social spending is slightly higher in *T*_*pre*_ than in *T*_*post*_. As a reminder, occupational exposure to foreign demand is derived from a sector-level variable that expresses the share of domestic value added that is embodied in foreign final demand. Substantively, the mean value of 0.35 thus refers to an occupation where, owing to its distribution across sectors, 35% of the weighted sectoral value added relies on foreign demand, with the other 65% relying on domestic demand. Personal work income refers to an individual's real yearly earnings in Swiss francs. The variable also shows a significant degree of variation, with the minimum value in particular being very low, something that one of the specifications in the robustness section will address. The university educated account for a third of all respondents, 12% are union members, exactly half are female and the vast majority (96%) are Swiss citizens.

**Table 1:** Descriptive statistics.

**Statistic**	***N***	**Mean**	**SD**	**Min**	**Max**
*T*_*pre*_ Soc exp support	921	0.45		0	1
*T*_*post*_ Soc exp support	921	0.41		0	1
*T*_*post*_ Exposure to foreign demand	921	0.35	0.12	0.05	0.61
*T*_*post*_ Satisfied with job	743	0.64		0	1
*T*_*post*_ University educated	921	0.33		0	1
*T*_*post*_ Union membership	921	0.12		0	1
*T*_*post*_ Personal work income	905	40,418	26,393	866	129,465
*T*_*post*_ Age in years	921	24.03	4.87	18	33
Female	921	0.50		0	1
Swiss citizenship	920	0.96		0	1

This table shows the native values for exposure and income. In the model, the former is entered after being standardized, the latter is logged and subsequently standardized.

### 3.7 Statistical model specifications

I use several statistical model specifications throughout the Results section below. The models that I use to test my main hypotheses are simple OLS (self-selection) and Linear Probability (socialization) models with year fixed effects (FE) that use the variables mentioned above. The first models that I present below are respectively random effects (RE) and random-effects-within-between (REWB) linear probability models. I use the latter to disentangle the respective contributions of *between-respondent* differences and *within-respondent* changes to the overall coefficient for occupational exposure to foreign demand (Bell and Jones, 2015; Bell et al., 2019). For any respondent *i*, *Between* variables are generated by taking the mean value ${\bar{x}}_{i}$ for any time-varying variable *x*, and *within* variables simply take the value $x_{it}-{\bar{x}}_{i}$. These models include year RE. I use linear probability models throughout the paper rather than logit models so that coefficients are more comparable across nested models. I also multiply the dichotomized DV by 100 in order for the coefficient to be directly interpretable as percentage points.

## 4 Results

### 4.1 Exposure and redistributive preferences among the working Swiss population

In this section, I start by exploring the relevance of occupational exposure to foreign demand for redistributive preferences among respondents to the SHP aged 30–65. In a first step, my goal is simply to replicate known results with respect to the negative association between exposure and redistributive preferences. In a second step, I use REWB models to disentangle the respective contributions of *between-respondent* differences and *within-respondent* changes to the overall coefficient for occupational exposure to foreign demand.

The left-hand side of Table 2 presents the results of the first step and shows, very simply, that more exposed individuals tend to be less supportive of social spending increases. The right-hand side of the table presents the results of three REWB models. It is immediately apparent from these models that when it comes to occupational exposure to trade among the Swiss population aged 30–65, only *between-respondent* differences contribute to the overall coefficient, as the *within* coefficient is completely indistinguishable from zero across all three models. This effectively confirms H3: among the population of older workers, changes in occupational exposure to foreign demand have no impact on redistributive preferences. This is all the more notable that models 4 and 5 show that within-respondent changes in household income do translate into changes in preferences, as does simply growing older. The *within* coefficient for union membership in model 5 barely falls short of being statistically significant at the *p* < 0.05 level, which suggests that among respondents who do join a union at some point, support for social spending is somewhat higher while they are actually union members, although the *between* coefficient is much more substantial. As far as the link between occupational exposure and social spending preferences is concerned, this leaves two possible mechanisms on the table that are equally compatible with these results and, again, not mutually exclusive: self-selection into occupations based on pre-existing preferences, and early labor market experience as a key site of preference formation with lasting effects. I investigate these in the following subsections.

**Table 2:** Linear probability models of support for social spending.

	***RE***		***REWB***		
	**Model 1**	**Model 2**	**Model 3**	**Model 4**	**Model 5**
			*Between variables*		
Exposure to foreign final demand	**−2.38** ^***^	**−2.24** ^***^	**−4.51** ^***^	**−4.57** ^***^	**−3.15** ^***^
	(0.32)	(0.34)	(0.46)	(0.46)	(0.50)
Age in years	**−0.24** ^***^	**−0.21** ^***^	**−0.25** ^***^	**−0.20** ^***^	**−0.21** ^***^
	(0.04)	(0.04)	(0.04)	(0.04)	(0.04)
HH decile income		**−0.46** ^***^		**−0.75** ^***^	**−1.02** ^***^
		(0.09)		(0.17)	(0.17)
Union member		**3.66** ^***^			**11.63** ^***^
		(0.57)			(1.28)
Public sector		**2.43** ^***^			**4.92** ^***^
		(0.56)			(1.08)
			*Within variables*		
Exposure to foreign final demand			−0.49	−0.45	−0.62
			(0.44)	(0.44)	(0.47)
Age in years			**−0.35** ^***^	**−0.35** ^***^	**−0.30** ^***^
			(0.08)	(0.08)	(0.08)
HH decile income				**−0.24** ^*^	**−0.26** ^*^
				(0.11)	(0.11)
Union member					1.25
					(0.64)
Public sector					0.52
					(0.67)
			*Time invariant variables*		
Female	**7.66** ^***^	**8.31** ^***^	**6.46** ^***^	**6.35** ^***^	**7.73** ^***^
	(0.81)	(0.83)	(0.83)	(0.83)	(0.85)
Swiss citizen		**−11.85** ^***^		**−11.29** ^***^	**−12.24** ^***^
		(1.17)		(1.14)	(1.17)
(Intercept)	**49.18** ^***^	**59.53** ^***^	**49.85** ^***^	**62.39** ^***^	**60.98** ^***^
	(2.00)	(2.32)	(2.33)	(2.63)	(2.71)
AIC	562, 417.54	516, 230.32	562, 383.75	558, 793.39	516, 124.36
BIC	562, 479.92	516, 327.41	562, 463.96	558, 900.26	516, 265.58
Log Likelihood	−281, 201.77	−258, 104.16	−281, 182.87	−279, 384.69	−258, 046.18
Num. obs.	54, 834	50, 306	54, 834	54, 497	50, 306
Num. groups: idpers	9, 014	8, 623	9, 014	8, 977	8, 623
Num. groups: year	15	15	15	15	15
Var: idpers (intercept)	1, 110.72	1, 070.56	1, 107.92	1, 088.32	1, 058.96
Var: year (intercept)	13.70	14.15	13.09	12.37	13.74
Var: Residual	1, 258.14	1, 264.86	1, 257.64	1, 257.46	1, 263.80

^***^p < 0.001; ^**^p < 0.01; ^*^p < 0.05. Coefficients that are statistically significant at the *p* < 0.05 level are in bold.

### 4.2 How important is self-selection?

I now go back to the main sample of 921 labor market entrants and explore the extent to which *T*_*pre*_ preferences are associated with occupational exposure to foreign demand in *T*_*post*_. New labor market entrants are obviously not randomly assigned to occupations, they instead make a series of choices that lead them down a particular occupational trajectory, and there are good reasons to assume that these choices are affected by attitudes and preferences inherited from their early socialization. Table 3 presents simple OLS models that regress occupational exposure to foreign demand in *T*_*post*_ on *T*_*pre*_ preferences as well as other potentially relevant factors, such as gender, university education, parental social class, *T*_*pre*_ household income decile and a dummy identifying whether the respondent lived in a German-speaking household in *T*_*pre*_. All models include year FE. For starters, model 6 shows that respondents who support increases in social spending in *T*_*pre*_ tend to end up in occupations that are less exposed by a magnitude of around a little over a fifth of a standard deviation. Model 7 adds a control variable for gender, which is shown to be a strong determinant of exposure in *T*_*post*_, as female respondents tend to end up in occupations that are less exposed by a magnitude of more than half a standard deviation. Because female respondents are stronger supporters of social spending increases, this model sees a notable decrease in the estimated impact of *T*_*pre*_ preferences on *T*_*post*_ exposure. The next three models add controls for university education, parental social class, *T*_*pre*_ household income decile and the language dummy. University education is shown to be associated with lower exposure in *T*_*post*_, and the children of unskilled workers are found to end up in less exposed occupations than children of members of the high-grade service class. Overall, these results tend to corroborate H2, as there appears to be a relatively robust link between *T*_*pre*_ preferences and *T*_*post*_ occupational exposure to foreign demand, indicating that a degree of self-selection is indeed happening. Finally, model 11 shows that self-selection actually only occurs among university educated respondents, which perfectly fits with the expectations formulated in H2a. As discussed above, this is likely the joint result of more firmly established preferences and more job opportunities, which enable university-educated entrants to pursue their preferred option.

**Table 3:** OLS models regressing *T*_*post*_ exposure to foreign demand on *T*_*pre*_ support for social spending.

	**Model 6**	**Model 7**	**Model 8**	**Model 9**	**Model 10**	**Model 11**
*T*_*pre*_ support for social spending	**−0.23** ^***^	**−0.13** ^*^	**−0.14** ^*^	**−0.14** ^*^	**−0.14** ^*^	−0.03
	(0.06)	(0.06)	(0.06)	(0.06)	(0.06)	(0.08)
*T*_*pre*_ support * University educated						**−0.33** ^*^
						(0.13)
*T*_*post*_ University educated			**−0.30** ^***^	**−0.30** ^***^	**−0.30** ^***^	−0.15
			(0.07)	(0.07)	(0.07)	(0.10)
**Parental social class (ref: high-service)**						
Low-service			−0.09	−0.06	−0.06	−0.05
			(0.09)	(0.09)	(0.09)	(0.09)
Small b. owner			0.14	0.16	0.16	0.16
			(0.12)	(0.12)	(0.12)	(0.12)
Skilled worker			−0.11	−0.09	−0.09	−0.09
			(0.08)	(0.08)	(0.08)	(0.08)
Unskilled worker			**−0.31** ^*^	**−0.30** ^*^	**−0.30** ^*^	**−0.30** ^*^
			(0.13)	(0.13)	(0.13)	(0.13)
*T*_*pre*_ Household income decile				0.01	0.01	0.01
				(0.01)	(0.01)	(0.01)
*T*_*pre*_ HH language German					0.00	−0.01
					(0.07)	(0.07)
Female		**−0.57** ^***^	**−0.57** ^***^	**−0.57** ^***^	**−0.57** ^***^	**−0.58** ^***^
		(0.06)	(0.06)	(0.06)	(0.06)	(0.06)
(Intercept)	−0.22	0.02	0.09	0.01	0.00	−0.02
	(0.19)	(0.18)	(0.19)	(0.21)	(0.22)	(0.22)
Year FE (*T*_*post*_)	Yes	Yes	Yes	Yes	Yes	Yes
R^2^	0.04	0.12	0.15	0.15	0.15	0.16
Adj. R^2^	0.02	0.11	0.13	0.13	0.13	0.14
Num. obs.	921	921	861	854	854	854

^***^p < 0.001; ^**^p < 0.01; ^*^p < 0.05. Coefficients that are statistically significant at the *p* < 0.05 level are in bold.

### 4.3 The impact of early occupational exposure to foreign demand on social spending preferences

In the previous section, I established that self-selection based on prior preferences does indeed occur and thus explains at least some of the association between occupational exposure to foreign demand and support for social spending among the working population. In this section, I test the article's main hypothesis, namely that exposure impacts preferences even when accounting for the impact of self-selection. The main results presented here are based on simple linear probability models of supporting an increase in social spending in *T*_*post*_. Table 4 explores a series of such models adding variables in a stepwise fashion. All models include year FE, to account for the possibility that some shock may unilaterally affect the preferences of all respondents at a given point in time. Model 12 shows that, among the baseline sample of new labor market entrants, a one standard deviation difference in occupational exposure to foreign demand is associated with the respondent being around 7.5 percentage points less likely to favor an increase in social spending. Model 13 adds a control for *T*_*pre*_ preferences, which are expected to be very strongly correlated with *T*_*post*_ preferences. The value associated with the exposure coefficient decreases meaningfully to around 5.5 percentage points, but stays decidedly negative and different from zero. Model 14 adds a control variable for logged personal work income, which somewhat surprisingly shows no association with support for social spending. Finally, model 15 adds a dummy identifying whether the respondent is university educated in *T*_*post*_, their age at that time, as well as whether the respondent is a union member. None of these control variables turn out to be significant predictors of support for social spending, and their addition hardly impacts the main coefficient of interest, which remains statistically significant at –4.95. These results lend strong support to H1, as they show occupational exposure to foreign demand to be a key determinant of how preferences for social spending of new labor market entrants evolve over the course of their early labor market experience. While *T*_*pre*_ support for social spending has been shown to predict *T*_*post*_ exposure in some capacity, these models show that *T*_*post*_ exposure impacts contemporary support for social spending even after accounting for pre-entry support.

**Table 4:** Linear probability models regressing support for social spending in *T*_*post*_ on exposure to foreign final demand in *T*_*post*_.

	**Model 12**	**Model 13**	**Model 14**	**Model 15**
*T*_*post*_ exposure to foreign final demand	**−7.58** ^***^	**−5.46** ^***^	**−5.57** ^***^	**−4.95** ^**^
	(1.68)	(1.59)	(1.62)	(1.64)
*T*_*pre*_ support for social spending		**33.51** ^***^	**33.13** ^***^	**32.86** ^***^
		(3.06)	(3.09)	(3.09)
*T*_*post*_ logged personal work income			8.05	−5.78
			(9.24)	(11.34)
*T*_*post*_ university educated				4.42
				(4.62)
*T*_*post*_ union member				−0.05
				(4.71)
*T*_*post*_ age in years				0.52
				(0.47)
(Intercept)	**51.25** ^***^	**33.93** ^***^	**29.10** ^**^	24.89
	(9.49)	(9.06)	(10.71)	(13.68)
Year FE (*T*_*post*_)	Yes	Yes	Yes	Yes
R^2^	0.05	0.16	0.16	0.16
Adj. R^2^	0.04	0.15	0.15	0.15
Num. obs.	921	921	905	905

^***^p < 0.001; ^**^p < 0.01; ^*^p < 0.05. Coefficients that are statistically significant at the *p* < 0.05 level are in bold.

**Figure 2:**
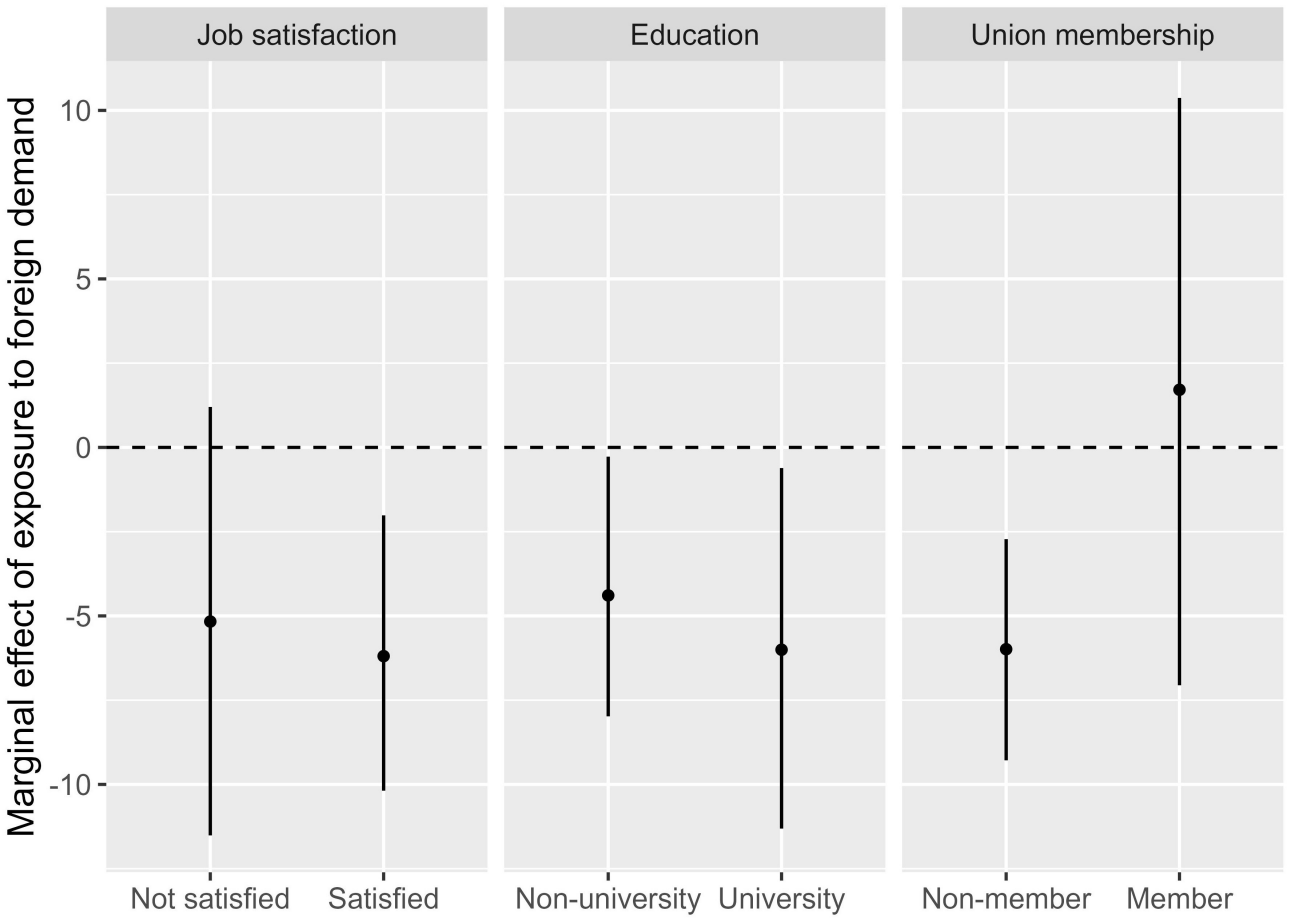
Marginal effect of exposure based on interaction models.

Next, I explore whether certain factors condition the main association of interest. Figure 2 presents conditional marginal effects of occupational exposure to foreign demand based on three interaction models (the full models are displayed in Supplementary Table S4). These interact the occupational exposure to foreign demand variable with dummy variables pertaining to job satisfaction, education and union membership. Considering the relatively small sample size, using such dummy interactions (rather than interacting exposure with other continuous variables) is likely to yield the most reliable results. The left panel explores whether respondents who are more satisfied with their job also respond more decisively to exposure. The job satisfaction variable is based on an item asking respondents to indicate their degree of satisfaction with their job in general on a scale from 0 (not at all satisfied) to 10 (completely satisfied). The variable skews heavily toward the higher values, so I code as satisfied those answering in one of the three highest response categories, which yields 265 non-satisfied and 462 satisfied respondents. The results of that first conditional model tend to corroborate hypothesis H1a, with the effect of exposure being negative and statistically significant for satisfied new labor market entrants, whereas it is more noisy among less satisfied respondents. The center panel reveals that whether the new entrant has university education or not hardly contributes to any differences in the effect of exposure, making it impossible to assess the relative validity of H1b and H1c, which yielded opposite expectations. This still constitutes an interesting result in regard of my findings about self-selection and university education. Even though university-educated have a high propensity to self-select into occupations based on pre-entry preferences, their preferences are just as affected by their early labor market experience as those of non-university educated entrants. This highlights the strength and relevance of the socialization mechanism at play. Finally, the right panel shows that the negative association between exposure and social spending preferences pertains primarily to non-union members. With only 12% of the sample being union members, it is not surprising that the estimated coefficient for this group is noisy, but it is nonetheless noteworthy that the point estimate for exposure is positive rather than negative. At the very least, this suggests that new labor market entrants who become union members do not buy into the international competitiveness argument to the same extent as their non-unionized counterparts.

### 4.4 Robustness

To assess the robustness of the results, I explore several alternative model specifications. All models presented in Table 5 replicate the baseline specification (Model 15), introducing targeted modifications to the criteria used for defining the final sample of new labor market entrants. These variations include lifting the restriction on being at once in one's initial training and actively occupied, adopting alternative age thresholds^4^, and implementing a minimum personal income threshold of CHF 10,000 in *T*_*post*_. Models 21 and 22 relax the conditions pertaining to being actively occupied in *T*_*post*_. The first retains individuals who have re-entered education in *T*_*post*_ after having initially left it in *T*, as long as they remain employed. The second includes all respondents meeting the entry criteria in *T*, regardless of activity status at *T*_*post*_, using their last job to define the occupational exposure variable^5^. Model 23 relies on the baseline sample and adds a control for public sector employment, at the cost of some 35 observations with missing data. Although my measure of exposure is defined at the occupational level and many occupations span both the public and private sectors—so that exposure is not inherently correlated with public sector employment—, some occupations prevalent in the public sector may be underrepresented in export-oriented sectors. It is therefore important to make sure that the results remain robust to the inclusion of this control. The core finding about higher exposure to foreign demand leading new labor market entrants to display lower support for social spending replicates across all alternative specifications. Regarding the results of models that include dropouts, these suggest that even for those new entrants who are out of the labor market in *T*_*post*_, the occupational characteristics of their last job remain relevant to their economic policy preferences, which makes sense insofar as they may consider it to be representative of their desired career path. Next, model 24 examines whether the results differ between respondents whose *T*_*post*_ preferences were measured in one of the 2001–2010 waves and those for whom the measurement occurred in one of the four later waves (2011, 2014, 2017, and 2020). The reference category is the latter, smaller group, and Year FE are dropped to avoid overfitting in the presence of the period dummy. The results are shown to hold across both groups of respondents.

**Table 5:** Linear probability models of support for social spending in *T*_*post*_.

	**Education**	**Age threshold**			**Income**	**Keep dropouts**		**Public sector**	**Cut-off**	**IPW**
	**Model 16**	**Model 17**	**Model 18**	**Model 19**	**Model 20**	**Model 21**	**Model 22**	**Model 23**	**Model 24**	**Model 25**
*T*_*post*_ exposure to foreign final demand	**−4.88** ^**^	**−5.71** ^**^	**−4.66** ^**^	**−4.71** ^**^	**−4.59** ^**^	**−5.65** ^***^	**−5.89** ^***^	**−3.88** ^*^	**−5.10** ^*^	**−4.16** ^*^
	(1.78)	(1.82)	(1.55)	(1.62)	(1.72)	(1.45)	(1.35)	(1.79)	(2.28)	(1.68)
*T*_*pre*_ support for social spending	**31.15** ^***^	**32.60** ^***^	**32.93** ^***^	**32.06** ^***^	**33.60** ^***^	**33.99** ^***^	**33.85** ^***^	**32.64** ^***^	**31.58** ^***^	
	(3.23)	(3.47)	(2.91)	(3.03)	(3.26)	(2.75)	(2.57)	(3.17)	(3.10)	
*T*_*post*_ logged personal work income	**−22.19** ^*^	−3.27	−0.73	−4.48	−12.58	−13.88	−1.80	−4.94	−2.10	
	(8.70)	(13.07)	(10.23)	(11.11)	(15.54)	(9.01)	(2.46)	(12.02)	(11.24)	
*T*_*post*_ university educated	8.15	5.66	6.47	4.26	7.36	4.00	3.92	5.54	5.00	
	(5.08)	(5.16)	(4.05)	(4.61)	(4.83)	(4.13)	(3.85)	(4.70)	(4.58)	
*T*_*post*_ union member	7.85	−2.96	−1.91	0.82	0.64	3.50	4.19	−1.67	−1.02	
	(6.47)	(5.33)	(4.33)	(4.66)	(4.87)	(4.35)	(4.09)	(4.81)	(4.74)	
*T*_*post*_ Public sector								4.22		
								(3.56)		
*T*_*post*_ age in years	0.55	0.24	0.01	0.52	0.20	0.52	0.15	0.44	0.57	
	(0.55)	(0.63)	(0.33)	(0.46)	(0.53)	(0.44)	(0.39)	(0.49)	(0.47)	
Post 2009									−0.12	
									(3.17)	
*T*_*post*_ exposure × Post 2009									−0.21	
									(3.25)	
(Intercept)	24.64	23.42	**35.48** ^**^	**25.97** ^*^	**35.72** ^*^	**27.02** ^*^	22.37	27.29	12.18	**39.96** ^***^
	(16.38)	(16.67)	(11.83)	(13.19)	(14.76)	(12.56)	(11.75)	(14.05)	(10.02)	(1.68)
Year FE (*T*_*post*_)	Yes	Yes	Yes	Yes	Yes	Yes	Yes	Yes	No	Yes
R^2^	0.15	0.17	0.16	0.15	0.16	0.17	0.16	0.16	0.13	0.01
Adj. R^2^	0.13	0.14	0.14	0.14	0.14	0.16	0.15	0.14	0.13	0.01
Num. obs.	853	725	1025	953	823	1129	1324	870	905	854

^***^p < 0.001; ^**^p < 0.01; ^*^p < 0.05. Coefficients that are statistically significant at the *p* < 0.05 level are in bold.

In addition, acknowledging that my continuous treatment variable (exposure) suffers from endogeneity with respect to the DV, an arguably more robust approach than controlling for *T*_*pre*_ preferences is to generate inverse probability weights (IPW) for said continuous treatment. The general idea behind this method is to use observed confounding variables to create individual propensity scores with respect to the treatment. I then use these propensity scores to generate IPW, which enables me to obtain an estimate of an average treatment effect (ATE) net of the influence of such confounding variables. The process for applying this method to continuous treatment was developed by Naimi et al. (2014) and I relied on the R implementation of the method described in Heiss (2020). The estimate that this method yields for the main association of interest falls quite close to that of model 15 and of the other models in the table, which should reassure us that these estimates do reflect the true effect of a one standard deviation in occupational exposure to foreign demand. Finally, Supplementary Table S5 replicates the models in Table 4 along with the IPW estimate, using a continuous rather than a dichotomized DV, which I also scale by 100 in order to bring the coefficients closer, although the latter cannot be interpreted as percentage points differences. The results are comparable across the first four models, whereas the main coefficient of interest slightly falls short of being statistically significant at the *p* < 0.05 level in the IPW model S10. Although model S10 only barely misses the *p* < 0.05 threshold, this suggests that exposure may be somewhat less decisive for the difference in preferences between “same” and “less” social spending than for the difference between “more” and the two lower response categories.

## 5 Conclusion

There have been numerous studies exploring the lasting negative consequences of unemployment spells during early adulthood. These show that individual attitudes as well as political behavior can be meaningfully impacted by an unfortunate start to one's labor market trajectory. The present article examines the other side of the coin, namely the impact of a specific occupational characteristic—exposure to foreign demand—on social spending preferences among those young adults who successfully integrated into the labor market after completing their initial training.

In the process, this article contributes several notable insights to the literature on trade exposure and export-dependence. First, it shows that individual social spending preferences prior to entering the labor market influence early occupational choices, confirming the self-selection hypothesis, particularly among university-educated new entrants. More importantly, it also shows that early labor market experiences play a crucial role in shaping an individual's economic policy preferences, even when accounting for pre-entry preferences. Specifically, labor-market entrants in occupations linked to export-dependent sectors tend to show reduced support for increases in social spending after several years on the job, irrespective of their pre-entry preferences. This holds true across all educational levels, although the effect appears to be most pronounced among individuals who report high job satisfaction and those not affiliated with unions. This result aligns with growing critiques of the contemporary relevance of the compensation hypothesis, and suggests that concerns about competitiveness are the driving force behind this relationship. Although the result is based only a small number of observations, the fact that union members do not appear to respond in the same way as other entrants remains a noteworthy finding, and warrants future research about the role of (different kinds of) labor unions in the socialization of new entrants to the labor market.

Taken together, my results provide empirical support for the notion that self-selection based on preferences and the adaptation of preferences during early labor market socialization are not mutually exclusive processes, as suggested by Kitschelt and Rehm (2014). The fact that there is a clear phenomenon of self-selection, but that the characteristics of an individual's occupation during her early labor market experience still have a significant impact on her preferences for social spending, is a remarkable result. It shows that even though prospective employees tend to self-select into occupations and sectors that match their initial preferences, their preferences are sensitive to the effects of workplace and organizational socialization. This particular finding travels well beyond the trade exposure literature and should be of interest to a wide range of scholars interested in the question of the interplay between an individual's occupation and her political attitudes. My results also suggest that the adaptation of preferences to one's occupational characteristics is not a lifelong process, but rather one that is specific to an individual's initial entry into the labor market. By contrast, for the population of working individuals aged 30 to 65, my results show, on average, no evidence of preference adjustment to changes in occupational characteristics. On the one hand, this lends additional support to the *impressionable years* hypothesis, which argues that political attitudes and behavior are highly malleable during early adulthood and become more rigid after a certain age. On the other hand, existing research has shown that older individuals do sometimes adjust their preferences or vote choice in response to comparable factors, such as the risk of their job being automated (Kurer, 2020). Future research could explore this apparent contradiction, in particular by examining the conditions under which individuals respond or do not respond to different types of changes in occupational characteristics. Is the magnitude of the change relevant—whether incremental or more radical? Is the broader economic context relevant to how individuals process such changes—whether the broader economy is booming or in crisis?

Indeed, the Swiss context may be relevant to the present findings. As a net exporter of goods and services, a significant proportion of Swiss GDP growth over the past decades is attributable to its export sector. Consequently, discussions on international competitiveness are common in both media and policymaking, meaning that new labor market entrants are likely already attuned to these issues, facilitating potential preferences adaptation once in a high exposure occupation. Additionally, Switzerland's strong economic performance, marked by relatively low unemployment compared to other Western European nations, may further reinforce competitiveness-oriented narratives. In light of the above, it would be interesting to determine whether these findings hold in countries that are less export-oriented or have experienced weaker labor market performance than Switzerland.

## Data Availability

Publicly available datasets were analyzed in this study. This data can be found here. For the ESS: https://www.europeansocialsurvey.org/. For the SHP: https://www.swissubase.ch/fr/catalogue/studies/6097/20179/datasets/932/3254/overview. For the OECD TIVA database: https://www.oecd.org/en/topics/sub-issues/trade-in-value-added.html.

## Footnotes

^1^A first random sample was drawn in 1999, with refreshment samples then drawn in 2004, 2013 and 2020.

^2^Nomenclature statistique des Activites economiques dans la Communaute Europeenne.

^3^The current SHP release includes ISCO08 codes for all years, whereas the early ESS waves still use ISCO88. I convert ISCO88 to ISCO08 using the DIGCLASS R package (Cimentada et al., 2023).

^4^Model 17 imposes stricter age limits, requiring respondents to be at least 19 years old in *T*_*post*_ and no older than 28 in *T*. In contrast, Model 18 retains the 18-year minimum in *T*_*post*_ but allows for labor market entry up to age 34. Model 19 removes the minimum age requirement altogether, thereby incorporating 32 minor respondents into the sample.

^5^See Supplementary Section 2 for a more detailed discussion pertaining to dropout respondents.
