# Supplementary material for: Early labor market experience and preferences for social spending: the impact of occupational exposure to foreign demand
Source: Front Sociol. 2025 Jun 18;10:1577352. doi: 10.3389/fsoc.2025.1577352 (PMC12217936; doi:10.3389/fsoc.2025.1577352)
Supplement: Supplementary file 1 [file Data_Sheet_1.pdf]

Supplementary Material

This document presents the supplementary material for 'Early labor market experience and preferences for social spending: The impact of occupational exposure to foreign demand'.

1 CONSTRUCTING THE INDIVIDUAL OCCUPATIONAL EXPOSURE VARIABLE

In order to construct my individual exposure variable, I draw from the empirical strategy of Baccaro and Neimanns (2022), who construct a variable perhaps best described as occupational trade exposure. Working on ISSP data – which only contains data on ISCO-88 occupations –, the authors take advantage of the fact that the European Social Survey precisely documents respondents' sectoral affiliation at the second level of aggregation as well as their occupation.[1] The way these authors proceed consists in calculating the distribution of ISCO-88 4-digits occupations across 15 sectors, and then effectively treating those probabilities as weights in order to assign to each 4-digits occupation a value that is the weighted combination of some quantity of interest measured at the sectoral level. Formally,

$$Occupational\ trade\ exposure_{io} = \sum_{s=1}^{N} P(s \mid o) * Sectoral\ trade\ exposure_s \quad \text{(S1)}$$

where i represents the individual, o the occupation, and s the sector. Using their occupation-level sectoral weights, Baccaro and Neimanns then calculate occupational exposure to trade as $\frac{Exports+Imports}{Output}$ for their ISSP respondents.

The way I proceed is similar, but differs on a couple of points. Most importantly, my procedure uses a 39-sector classification[2], which allows me to capture, among other things, the diversity of manufacturing in terms of the quantity of interest.

In order to estimate the probability that a given occupation is active in a given sector in my target country of Switzerland, I use a sample of 25 countries in the ESS surveys that I call source countries, for which I separately calculate the probability that a given occupation is active in a given sector based on the data. I then apply corrections based on the relative sizes of all sectors for all source country-Switzerland country-pairs, using number of respondents from the source countries as weights, since probabilities derived from a sample of 15000 are more reliable than those from a sample of 3000. Once this is done, I ensure that the probability mass function of a given occupation always sums to 1 across all sectors for Switzerland.

The equation that details the method described above is the following:

[1] More specifically, ESS 1 refers to NACE rev1, ESS 2-4 to NACE rev1.1, and finally ESS 5-9 refer to NACE rev2.

[2] Note that the first four waves of ESS are based on revisions 1 and 1.1 of the Statistical classification of economic activities in the European Community (NACE), whereas waves 5 through 9 as well as TIVA 2021 use revision 2. To deal with this issue and nonetheless incorporate the first four ESS waves in my analyses, I use an OECD conversion table published in Horvát and Webb (2020, 44). Since the ESS sector variable is highly fine-grained, it is possible to maintain a high degree of consistency while operating this conversion.

$$P(sec_j^{CH} \mid occ_i^{CH}) = \sum_{c=1}^{C} w_c \frac{P(sec_{cj} \mid occ_{ci}) * \frac{sec_j^{CH}/GDP^{CH}}{sec_{cj}/GDP_c}}{\sum_{j=1}^{J} P(sec_{cj} \mid occ_{ci}) * \frac{sec_j^{CH}/GDP^{CH}}{sec_{cj}/GDP_c}} \tag{S2}$$

where j indexes sectors, i indexes occupations and c indexes source countries, and w_c represents the normalized weights based on the total number of respondents by country. The concrete procedure starts with a dataset of country-sector-occupation observations, with a variable that measures the proportion of individuals in a given ISCO-08 4-digits occupation employed in a given sector. I call this variable `occu_sect`. Note that ESS post-stratification weights were applied systematically.

Separately, I generate a dataset that, for each sector in each source country, lists the relative importance of that sector in Switzerland. I do so first for each individual year between 2002 and 2020 and then take the mean of that ratio across all years, I call this variable `ratio_sect`. By definition, `ratio_sect` takes a value of 1 when source country and target country – Switzerland – are one and the same.[3]

The `occu_sect` variable is then multiplied by the `ratio_sect` variable, which acts as a correction factor of sorts. I then effectively have 25 estimates of the probability for a given occupation to be active in a given sector in Switzerland, based on observations in each 25 source countries, to which the `ratio_sect` correction factor was applied. From here, I take the weighted mean of those 25 source country estimates, with the weighting factor being the total number of respondents from each country that are being used for this exercise, namely those that have no missing values on either ISCO or their sector. As a last step, I ensure that the probability mass function of a given occupation always sums to 1 across all sectors, i.e. that it is effectively on the probability scale.

In order to still account for individuals that gave an imprecise response regarding their occupation, the main procedure involving ISCO-08 4-digits is repeated for 3-digits, 2-digits and 1-digit. While the information from those respondents with valid ISCO-08 4-digits is used to generate the sectoral weights for the less precise ISCO-08 measures, these more vague measures only come into play in the final measure in the absence of more precise information on the respondent's occupation.

2 RESPONDENTS DROPPING OUT OF THE MAIN SAMPLE BETWEEN T AND T_{post}

One of the conditions for a respondent to be part of the main sample is to be actively occupied in T_{post}, as defined with respect to labor market entry. Out of the 1354 entrants in T, only 921 remain in T_{post} as per this criteria. Among the dropouts, 40 are unemployed, 70 exited the labor force for unspecified reasons, 91 exited the labor force to go back to studying full time, and 226 are actually actively occupied, but have also gone back to studying, so that applying the main criteria leads them to be excluded.

While models 21 and 22 of table 5 in the main text already show that the inclusion or exclusion of dropouts from the sample does not impact the results, it can be interesting to explore whether dropping out is in some way associated with occupational exposure at the time of entry into the labor market, which could imply that differential attrition with respect to the IV of interest impacts the results in some way. Table S1 shows that this is not the case, as occupational exposure in T has no predictive power on whether the new entrant is still active in T_{post}. Table S2, by contrast, does show that respondents who drop out of the sample hold slightly more favorable views vis-à-vis social spending, in T_{pre} as well as in T_{post}, but the

[3] Considering that the way in which a specific ISCO-08 4-digits occupation is distributed across sectors is stable over time is of course a simplification, but a reasonable one to make over a period of less than 20 years.

Table S1. Linear probability models of being actively occupied in T_{post} following entry in T

	Model S1	Model S2
T Exposure to foreign final demand	0.01	0.02
	(0.01)	(0.01)
(Intercept)	$\mathbf{0.69}^{***}$	$\mathbf{0.78}^{***}$
	(0.01)	(0.08)
Year FE (T_{post})	No	Yes
R^2	0.00	0.01
Adj. R^2	0.00	0.00
Num. obs.	1320	1320

$^{***}p < 0.001$; $^{**}p < 0.01$; $^{*}p < 0.05$

Table S2. Share in favor of more social spending

	Dropouts	Non-dropouts
T_{pre}	51.3	45.2
T_{post}	47.3	40.7

two groups move in very similar ways between the two periods, which again make it difficult to imagine that it could impact the results.

3 ADDITIONAL TABLES AND FIGURES

Figure S1. Histograms of effective absolute distances of T_{pre} and T_{post} to T.

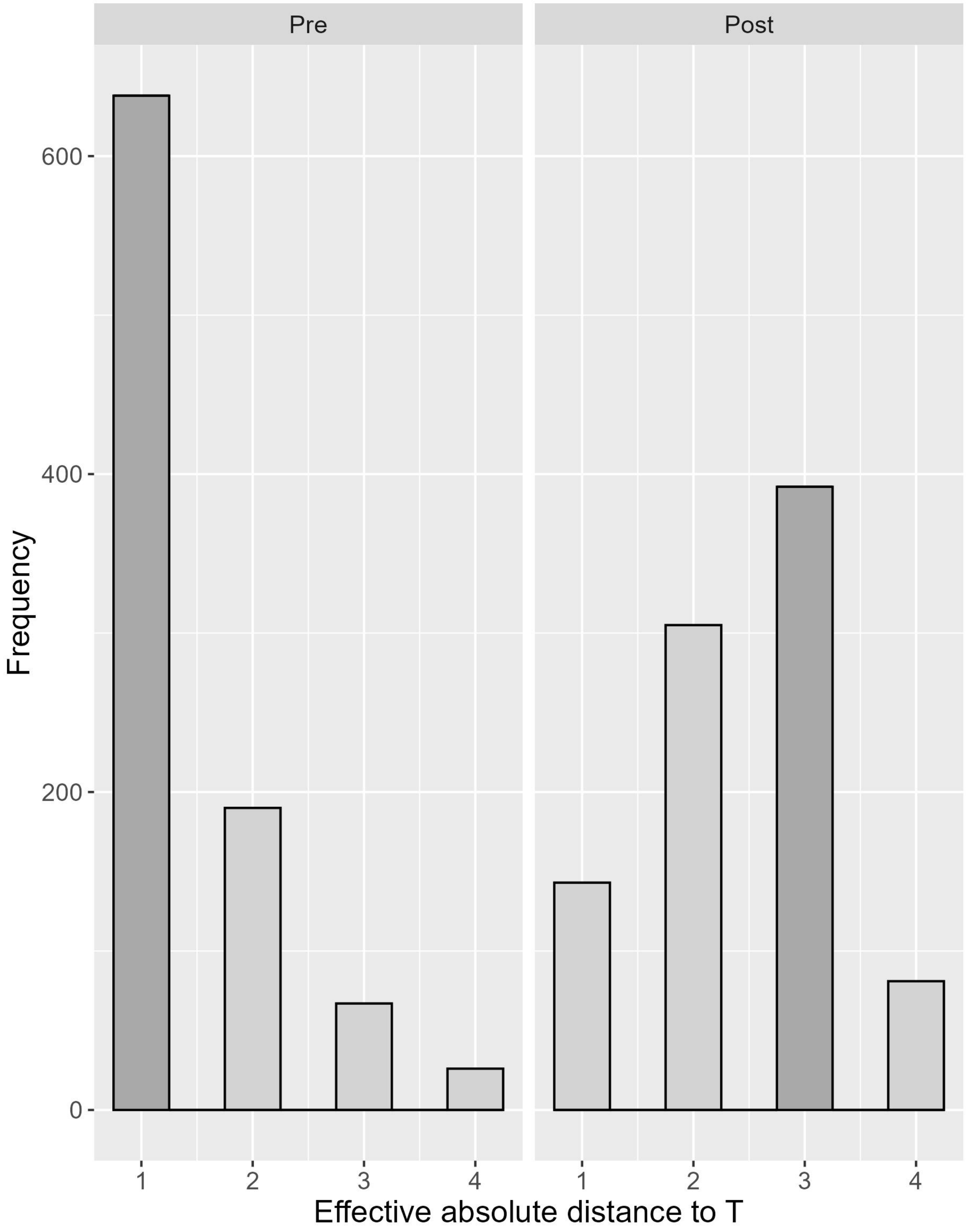

Table S3. Distribution of T and T_{post} years in final sample

Year	2000	2001	2002	2003	2004	2005	2006	2007	2008	2009	2010	2011	2012	2013	2014	2015	2016	2017	2018	2019	2020
T	149	58	52	36	38	59	54	75	71	63	39	28	34	29	15	23	16	15	38	29	
T_{post}		26	37	115	54	40	43	41	62	109		139			97			64			94

Due to measures of preferences becoming scarcer from 2011 onward, cases that are suitable with respect to T_{pre}, T and T_{post} become rarer, something that is to be expected.

Table S4. Linear probability interaction models of support for social spending in T_{post}

	Model S3	Model S4	Model S5
T_{post} Exposure to foreign final demand	-5.09	$\mathbf{-4.29}^{*}$	$\mathbf{-6.04}^{***}$
	(3.15)	(2.06)	(1.76)
T_{post} Exposure * Job satisfaction	-1.15		
	(3.78)		
T_{post} Exposure * University educated		-1.79	
		(3.41)	
T_{post} Exposure * Union member			7.82
			(4.63)
T_{pre} support for social spending	$\mathbf{32.03}^{***}$	$\mathbf{32.80}^{***}$	$\mathbf{32.86}^{***}$
	(3.44)	(3.10)	(3.09)
T_{post} Logged personal work income	-1.65	-5.63	-4.96
	(12.83)	(11.35)	(11.34)
T_{post} University educated	1.06	4.03	4.85
	(5.25)	(4.68)	(4.62)
T_{post} Union member	-0.97	-0.32	1.47
	(5.42)	(4.74)	(4.79)
T_{post} age in years	0.77	0.55	0.50
	(0.55)	(0.47)	(0.47)
Job satisfaction	-3.54		
	(3.54)		
(Intercept)	7.46	24.50	23.94
	(13.69)	(13.70)	(13.68)
Year FE (T_{post})	Yes	Yes	Yes
R^2	0.17	0.16	0.17
Adj. R^2	0.15	0.15	0.15
Num. obs.	727	905	905

$^{***}p < 0.001$; $^{**}p < 0.01$; $^{*}p < 0.05$

Table S5. OLS models of support for social spending in T_{post} (continuous)

	Model S6	Model S7	Model S8	Model S9	Model S10
T_{post} Exposure to foreign final demand	$\mathbf{-10.86}^{***}$	$\mathbf{-7.52}^{**}$	$\mathbf{-7.57}^{**}$	$\mathbf{-5.96}^{*}$	-5.29
	(2.82)	(2.66)	(2.71)	(2.74)	(2.78)
T_{pre} support for social spending		$\mathbf{36.40}^{***}$	$\mathbf{35.91}^{***}$	$\mathbf{35.07}^{***}$	
		(3.25)	(3.28)	(3.27)	
T_{post} Logged personal work income			16.69	-20.28	
			(15.49)	(18.94)	
T_{post} University educated				8.42	
				(7.73)	
T_{post} Union member				3.26	
				(7.86)	
T_{post} age in years				$\mathbf{1.60}^{*}$	
				(0.79)	
(Intercept)	$\mathbf{219.36}^{***}$	$\mathbf{132.31}^{***}$	$\mathbf{123.11}^{***}$	$\mathbf{108.82}^{***}$	$\mathbf{212.24}^{***}$
	(15.91)	(16.82)	(19.35)	(23.93)	(2.80)
Year FE (T_{post})	Yes	Yes	Yes	Yes	Yes
R^2	0.04	0.16	0.15	0.17	0.00
Adj. R^2	0.03	0.14	0.14	0.15	0.00
Num. obs.	921	921	905	905	854

$^{***}p < 0.001$; $^{**}p < 0.01$; $^{*}p < 0.05$
